# How to motivate planners to participate in community micro-renewal: An evolutionary game analysis

**DOI:** 10.3389/fpsyg.2022.943958

**Published:** 2022-08-22

**Authors:** Dong Wang, Meiling Wu, Jiulong Qu, Yuncui Fan

**Affiliations:** ^1^School of Business and Management, Jilin University, Changchun, China; ^2^School of Economics and Management, Jilin Jianzhu University, Changchun, China

**Keywords:** community micro-renewal, urban renewal, community planner, evolutionary game, stakeholders

## Abstract

In China, grassroots governments have attempted to introduce community planners into community micro-renewal, using their expertise to guide the design and implementation of community micro-renewal. However, issues remain to be studied and resolved, including how to effectively play the community planner role to coordinate multiple parties’ interests. This study constructed an evolutionary game model based on the behaviors of multiple subjects with participation by community planners, to establish the payment functions on both sides of the game under different choice strategies; explore evolutionary stabilization strategies by replication dynamic equations; and to analyze the conditions for the multi-party evolutionary game to reach the ideal stable state. The findings: (1) Show that financial subsidies provided by the grassroots government to community planners have a positive effect on the latter’s behavioral choices; (2) Illustrate the path of the tripartite evolutionary game among the grassroots government, residents, and community planners to reach ideal stability (incentive, active participation, and positive promotion); and (3) Describe how the project benefits from community planners promoting community micro-renewal can effectively promote their positive behavioral choices.

## Introduction

China has undergone a process of rapid urbanization following its reform and opening-up (Lin and Zhu, 2021); however, local governments’ over-attention to the speed of development has led to problems like “spread pancakes.” Although urbanization is increasing, the lack of investment in management and maintenance of older metropolitan areas has led to difficulties with the infrastructure meeting residents’ needs (Zhang et al., 2021), and consequently to accelerated aging of community resources and impoverished living conditions. In 2015, the Central Urban Work Conference proposed adhering to the people-centered development principle, which focuses on improving sustainability and livability in urban development, promoting transformation of urban development from extensive growth to connotative development, and clarifies “urban repair” policy. Within this context, local governments have begun focusing on urban organic renewal, one essential aspect of which is community micro-renewal.

Compared with urban renewal at the macro level, community micro-renewal appears in the context of stock upgrading and development. Its prominent feature is being “people-oriented” (Williamson and Ruming, 2019)—including improving community infrastructure and environment—with a design mechanism of resident participation in organization and management, and the cultivation of collaborative community governance. Community micro-renewal is a “bottom-up” professional approach aimed at meeting the needs of multiple subjects. However, though many older communities need such renewal and transformation, a lack of professional guidance has led to disorderly community renewal and a low degree of active participation by community residents. These problems have also attracted the attention of planners who are interested in social welfare (Hosseini et al., 2017).

In the Hierarchical organizational environment, urban renewal is a top-down process led by the government (Liu et al., 2020), in which the needs and opinions of community residents are easily ignored, likely leading to wasted resources and social conflict (Zhao G. et al., 2021). In contrast, community micro-renewal is usually a bottom-up process led by the residents themselves. In this case, the government takes a supervisory and guiding role (Zheng et al., 2020). When community planners participate in community micro-renewal, they can leverage their professional advantages and coordination functions to not only help the government guide community micro-renewal, but to identify and meet residents’ needs.

Although some cities have implemented the community planner system, and some micro-renewal cases have been led by community planners, trust and participation among community residents in such processes are insufficient and the scope of community planning is immature. Exploring the institutional system of community planners’ participation in community micro-renewal within China’s national context is thus essential. The rise of grassroots planners not only means that planning has moved beyond a government policy tool for shaping urban space “from top-to-bottom,” it is also a form of social collaborative governance. Planners have become important coordinators of the relationship between the government, residents, and participating enterprises; they pay greater attention to “communication” and “coordination” in the planning process, and emphasize tracking and response by grassroots-constructed problems and the initiation of social participation. Therefore, this article addresses three questions:

(1) What approach should the government take to enhance the role of community planners?
(2) What strategies should be adopted by the government, community residents, and community planners to optimize the effectiveness of community micro-renewal?
(3) What are the principles of participation and design for a collaborative mechanism of community micro-renewal stakeholders?

## Literature review

### From urban renewal to community micro-renewal

Urban regeneration is an important strategic urban development approach (Zhang et al., 2021) that can make effective contributions to cities’ economic development and cultural heritage (Campbell et al., 2017). Following World War II, urban construction shifted from large-scale knock-down and redevelopment to community renewal focused on state welfare (Hackworth and Smith, 2001; Ferretti and Grosso, 2019). At this time, the government began implementing renewal policies that considered the rational allocation of public resources and improved older communities (Couch et al., 2011). However, it is often difficult to attract market investment in areas with decaying physical infrastructures and high concentrations of impoverished residents (Weber, 2010). Such residents also suffer losses in urban regeneration, due to constraints on their participation and opportunities (Ng, 2002; Díaz Orueta, 2007). With advances in urban regeneration research, such approaches began to promote civic power, focusing on the roles of local community leaders and non-government organizations (NGOs) in these processes (Hemphill et al., 2004; Rossi, 2004). Government policies have since provided incentives for developers to participate in regeneration, creating an impetus for district regenerations.

In the era of stock development, urban regeneration sustainability cannot rely on the government alone. Rather, it needs participation by the market, social forces, and community residents. According to this approach, government involvement in the urban renewal process is reduced (Bailey, 2012) and there is greater involvement by the private sector and other NGOs, which support and monitor implementation of urban renewal across the decision-making and implementation processes (Kim and Jang, 2017; Vale, 2018). Multiple-driven urban regeneration has become the main model (Hastings, 1996), maintaining the urban fabric and spatial patterns of historic areas and original communities, and adapting to new spatial requirements through physical transformation or cultural activities (Steinberg, 1996). Thus multiple-driven regeneration is often characterized by large-scale, high-risk and commercial–public attributes (Adams and Hastings, 2001; Fainstein, 2008). Such regeneration targets are also characterized by multiple demands for functional improvements and complex interests. Social-based solutions are increasingly an innovative way for foreign scholars to reconcile the stakeholders involved in the urban renewal process (Wrigley et al., 2002; Díaz Orueta, 2007; Sasaki, 2010).

In the process of urban renewal, the disadvantages of large-scale demolition and reconstruction have emerged gradually. Wasted urban resources is a major problem, as is destruction of cities’ organic nature and diversity; both of these factors seriously affect preservation and inheritance of urban culture (Jacobs, 2021). One solution to reducing waste is transforming some older buildings (Ferretti and Grosso, 2019); to achieve this, scholars have proposed that the government unite community forces to promote community regeneration (Gorczyca et al., 2020), emphasizing humanism and highlighting urban cultural values (Vale, 2018; Williamson and Ruming, 2019).

In both theory and practice, the concept of progressive community renewal has received greater respect when applied at the smaller scale. Now, scholars have begun focusing on the multidimensional renewal of humans’ living environment, advocating for sustainable urban development (Park, 2014) and transformation from material-centered urban renewal to people-centered community micro-renewal (Terry and Townley, 2019). Greater numbers of scholars have begun conducting research on community regeneration issues at the urban micro-level (Perez et al., 2018), focusing not only on the construction and transformation of the physical environment but on social, economic, ecological, and cultural transformations, emphasizing how to improve residents’ living conditions and enhance inclusiveness (Niu et al., 2018). The regeneration approach has also become more affluent, ranging from urban-scale spatial optimization and industrial upgrading to community-scale overall transformation of older residential areas and urban villages, while focusing on community environment improvements (Brown, 2017).

### Community planners and community micro-renewal

In 2020, more than 14 million households in China required community renewal (Chen et al., 2021). In 2021, the Government Work Report proposed to newly renovate 53,000 older urban communities. While the government improves the urban landscape and promotes rapid urban development through community renewal (Verdini, 2015), a more prominent role for resident participation in community renewal is also needed (Cui et al., 2018). Residents’ preferences and behaviors significantly impact government decisions (Bromley et al., 2005). Therefore, encouraging residents to participate in community micro-renewal is crucial. However, the current major urban renewal model in China is top-down, and residents are accustomed to abiding by governmental decisions; thus, they rarely participate in renewal projects (Li et al., 2014). The community micro-renewal focus is on personal interests, which affects its promotion. Community planners can act as a communication bridge between the government and public, to strengthen residents’ awareness of community wholeness through comprehensive outreach and training, encouraging active and enthusiastic participation (Tu et al., 2021).

The community planner system can be traced back to the 1960s when it emerged within community planning development in Europe and the United States. It was discovered that community planners could effectively facilitate information communication among multiple subjects and promote the role of community residents in urban planning. In China, Shenzhen was the first city to promote the community planner system, after which a large number of urban renewal cases involving community planners were implemented in Beijing (Liu Jiayan, 2021), Shanghai (Tu et al., 2021), Chengdu (Zhao et al., 2021b), and Shenzhen (Sima et al., 2020), as shown in Table 1. Many successful cases have demonstrated the feasibility and necessity of implementing the community planner system in China (Cao et al., 2022). Yet this system remains at an early developmental stage. Due to differences in economic development, resources, cultures, and urban characteristics, it can be challenging to match residents’ participation demands to the actual conditions (Zhao et al., 2021a; Cao et al., 2022). Community planners must still clarify their role and improve their influence over both public and social forces, as the government remains the leading force in promoting the community planner system. Unlike NGO participation in community micro-renewal in Europe and the United States, Chinese non-profit organizations generally lack funding (Zhiyuan, 2016), and it remains difficult for NGOs to take the lead in community micro-renewal.

**TABLE 1:** Job descriptions among community planners participating in community micro-renewal.

City	Job description	Community
Beijing	(a) Investigation and survey, (b) Communication, (c) Technical consultation, (d) Planning evaluation, (e) Summary publicity.	Chaoyangmen Community, Dongcheng District
Shanghai	(a) Develop community assessment methods, (b) Understand residents’ needs, (c) Complete community planning, (d) Guide community physical construction, (e) Decompose and implement system planning.	North Sichuan Road Community, Hongkou District
Chengdu	(a) Volunteer, (b) Interpret residents’ needs, (c) Complete the design scheme, (d) Community public space construction.	Yulin East Road Community, Wuhou District
Shenzhen	(a) Preparation of community plans, (b) Community building management, (c) Coordination of subject relations, (d) Arrange planning implementation.	Guihua Community, Longhua District

Through analyses of existing research and practice cases, it is evident that community planners play important roles in community micro-renewal. Yet most studies have been based on the assumption that community planners participate objectively—and have rarely considered them as rational actors with individual interests, and potential conflicts of interest. When choosing whether to provide services to community residents, community planners may consider the potential for future material or spiritual benefits. If they fail to realize the expected benefits, they may choose not to participate, or become passive even when vigorously promoted by the government. Thus, further implementation of the community planner system requires understanding the mechanisms of planners’ behavioral choice strategies. With such information, corresponding incentive measures can be formulated to encourage their active and continuous participation in community micro-renewal.

## Stakeholders and assumption

### Behavioral strategies of core stakeholders

#### Community residents

Community residents are both the beneficiaries of community micro-renewal projects and the decision-makers and supervisors in the implementation process. Therefore, the behavioral decisions of community resident groups significantly influence the implementation and promotion of community micro-renewal projects.

Because of varying interests within resident groups, their behavioral strategies are expressed in two ways: active participation or passive participation. When community residents feel that the community micro-renewal benefits outweigh the inputs, they often choose to actively participate in the project. This can manifest as active support, cooperation, participation, and active supervision of project implementation. However, when they feel that the input for community micro-renewal is unnecessary or outweighs the benefits, they tend to take a negative participation strategy. This usually manifests as lack of ownership, insensitivity to community problems, inactive participation in the supervision of project implementation, and not actively maintaining the project results.

#### Grassroots government

In China, grassroots governments still play an essential role in community micro-renewal projects, as decision-makers, guides, coordinators, and administrators. They are needed to guide resident groups, enterprises, and community planners to participate in community micro-renewal projects through specific administrative, legal, or economic means, to promote project effectiveness. Therefore, the grassroots government is a vital force in promoting community micro-renewal and is an essential guarantee of innovative community governance for achieving community sustainability and long-term development.

Grassroots governments have two behavioral strategies: incentive or no incentive. Community micro-renewal can generate social benefits and reflect the performance of grassroots governments, so some are willing to adopt incentives to effectively promote community micro-renewal. However, many community micro-renewal projects in China are still in the initial development stage. There is not yet a mature, systematic program, leaving development prospects particularly uncertain. Given the risks and costs, some governments take a conservative, passive attitude and choose not to incentivize.

#### Community planners

Community planners have two behavioral strategies: aggressive advancement and non-advancement. Community planners who adopt an aggressive advancement strategy are influenced by government incentives, academic achievements, social reputation, and personal values. However, community planners have limited knowledge of the community itself, which may create a conflict between professionalism and localism. It is difficult to advance a project, requiring significant time and energy. In this case, the community planner may also adopt a negative promotion strategy.

Personal sentiments usually drive community planners to participate or consult part-time. There is no mature system to protect the rights and interests of community planners in community micro-renewal projects. Thus, overall, community planners’ willingness to participate is low, and it is generally challenging to promote community micro-renewal in public communities. Simultaneously, the government will pay a specific cost to improve the system or reward community planners to motivate their participation. Therefore, whether community planners actively promote community micro-renewal and whether the government encourages it becomes a behavioral game.

### Assumptions and parameters

Based on these cumulative analyses, this article proposes several assumptions:

(1) The tripartite subjects of grassroots government, resident groups, and community planners are all finitely rational. Their behavioral strategies are constantly learning and adjusting, under objective conditions, to pursue benefit maximization of their behavioral decisions.

(2) The behavioral strategies of the grassroots government are (incentive, no incentive) with the probability of occurrence (μ, 1−μ); the behavioral strategy of the resident groups are (active participation, negative participation) with the probability of occurrence (*v, 1-v*); and the behavioral strategy of the community planners are (active promotion, no promotion) with the probability of occurrence (ρ,1−ρ).

(3) When the grassroots government encourages community planners to lead community renewal planning, in addition to the corresponding project funds *C9*, they must invest in economic subsidies *K* to encourage community planners. When the grassroots government does not encourage community planners to lead the community micro-renewal, the grassroots government conducts the community renewal and transformation according to the traditional project model. There are no community planners to plan, regulate, promote, coordinate or guide residents to participate in community renewal. The poor effects of community renewal and residents’ conflicts occur frequently, or residents’ negative participation conflicts with the government. The credibility and image of the grassroots government are damaged, *C10*. When the government takes incentive measures to jointly encourage community planners and residents to promote community micro-renewal, performance improvement will be rewarded by the higher authorities, *W3*.

(4) The vested interests of residents who have not yet undertaken community micro-renewal is *R11*. If residents choose to participate actively, they will cooperate with the grassroots government in the micro-renewal process, maintain effective communication with the community planner, and fully express their needs and renewal goals. In this case, the residents’ group will contribute a certain amount of money and effort; they will also risk taking responsibility, which is assumed to be the participation cost *C11*. In addition, the residents’ group will be rewarded by the grassroots government for their active participation in the community micro-renewal *R12*, which brings a sense of psychological achievement as the main body of community affairs management *R13*. The residents’ active involvement in the community micro-renewal will also improve their living environment and quality of life *R14*. If residents passively participate in community micro-renewal and ignore the scheme design and project implementation, the renewal may not meet their needs, and they will bear certain losses *D2*; if they have conflicts with the community planner, the latter will also bear losses *D3*.

(5) When community planners adopt a strategy of not promoting community micro-renewal, they do not actively participate in planning the renewal of older communities and instead carry out traditional projects to gain revenue, *R15*. When community planners adopt an active promotion strategy, they assume the role of planner, advocate, supervisor, and coordinator and bear the costs of participation, *C12*; simultaneously, community planners will gain financial income from participating in community micro-renewal *R16*, project experience and competence enhancement *R17*, and personal satisfaction *R18*.

(6) If community planners do not actively promote community micro-renewal, residents remain passive, and grassroots government does not take incentive measures, there will ensure a deteriorating living environment, causing dissatisfaction among residents; this, in turn, will have negative social effects and the grassroots government will suffer corresponding punishment from higher authorities and loss of reputation *B1*.

**FIGURE 1:**
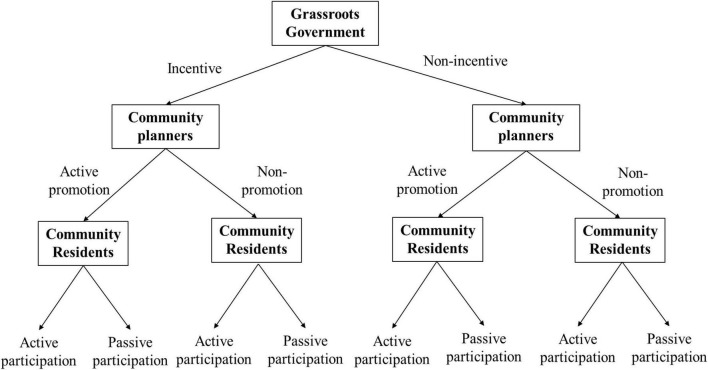
Community micro-renewal evolutionary game subjects and strategy choices.

According to these strategies, sets of evolutionary games by grassroots government, residents, and community planners, there are eight strategy combinations in the community micro-renewal game system. The tripartite evolutionary game tree model is shown in Figure 1.

The parameters of community micro-renewal game subjects are shown in Table 2.

**TABLE 2:** Parameters.

Parameter	Description
*C9*	Project funds invested by grassroots government
*C10*	Damage to the credibility and image of grassroots government
*C11*	Resident participation costs
*C12*	Community planner engagement costs
*K*	Financial subsidies for community planners provided by grassroots government
*W3*	Grassroots government performance improvement rewarded by higher authorities
*R11*	Residents’ vested benefits
*R12*	Residents’ active participation rewarded by grassroots government
*R13*	Residents’ sense of achievement from participating in community management
*R14*	Additional benefits for residents from improved livelihoods
*R15*	Community planners involved in general projects gain
*R16*	Community planner income from community micro-renewal project
*R17*	Project experience and capacity-building for community planners
*R18*	Personal emotional satisfaction for community planners
*D2*	Resident losses from community micro-renewal not meeting targets
*D3*	Lack of resident cooperation leading to loss of community planners
*B1*	Loss of grassroots government from penalties imposed by higher authorities
μ	Probability of choice of incentive strategy by grassroots government
*v*	Probability of residents choosing to actively participate
ρ	Probability of community planners choosing to actively promote

## Evolutionary game model

### Payout matrix and modeling

Based on the model parameter assumptions, Tables 3, 4 show the payoff matrix when the grassroots government chooses to incentivize or not to incentivize the two strategies, respectively.

**TABLE 3:** Tripartite payoff matrix under grassroots government incentives (μ).

Game subjects	Community planners	
	
Residents	Active promotion(ρ)	No promotion(1−ρ)
Active participation(*v*)	*W*3−*C*9−*R*12 − *K*, *R*11 + *R*12 + *R*13 + *R*14 − *C*11,*K* + *R*16 + *R*17 + *R*18 − *C*12	*W*3 − *C*9 − *C*10 − *R*12, *R*11 + *R*12 + *R*13 + *R*14 − *C*11,*R*15
Negative participation (1−*v*)	*W*3−*C*9−*C*10−*K*, *R*11+*R*14−*D*2, *K* + *R*16+*R*17+*R*18−*C*12−*D*3	−*C*9−*C*10, *R*11+*R*14−*D*2, *R*15

**TABLE 4:** Tripartite payoff matrix under grassroots government non-incentive (1-μ).

Game subjects	Community planners	
	
Residents	Active promotion(ρ)	No promotion(1−ρ)
Active participation(*v*)	−*C*9, *R*11+*R*13+*R*14−*C*11, *R*16+*R*17+*R*18−*C*12	−*C*8−*C*10, *R*11+*R*13+*R*14−*C*11, *R*15
Negative participation (1−*v*)	−*C*9−*C*10, *R*11+*R*14−*D*2, *R*16+*R*17+*R*18−*C*12−*D*3	−*C*9−*C*10−*B*1, *R*11−*D*2, *R*15

The expected benefits of the incentive strategy adopted by grassroots government are:


(1) $$U_{\mu }=\rho v\left(W3-C9-R12-K\right)+\rho \left(1-v\right)(W3-C9-R12$$

-K)+(1-ρ)v(W3-C9-C10-R12)+(1-ρ)

(1-v)(W3-C9-C10)=ρv(R12)+ρ(C10-R12

-K)+v(-R12)W3-C9-C10

The expected benefits of a no-incentive strategy for grassroots government are:


(2) $$U_{1-\mu }=\rho \,v\,\left(-C\,9\right)+\rho \,\left(1-v\right)\,\left(-C\,9-C\,10\right)+\left(1-\rho \right)$$

v⁢(-C⁢9-C⁢10)+(1-ρ)⁢(1-v)⁢(-C⁢9-C⁢10-B⁢1)

=ρ⁢v⁢(C⁢10-B⁢1)+ρ⁢B⁢1+v⁢B⁢1-C⁢9-C⁢10-B⁢1

Thus, the average benefits expected by grassroots governments choosing a mix of incentive and no incentive strategies are:


(3) $${\bar{U}}_{\mu }=\mu \,U_{\mu }+\left(1-\mu \right)\,U_{1-\mu }$$

According to the Malthusian dynamic equation, when the payoff of a strategy chosen in a game problem is higher than the average payoff of the other strategies, the strategy is considered able to adapt to the evolutionary process of the group and has strong resistance to intrusion by mutating strategies (Friedman, 1991); the replication dynamic equation for grassroots government is:


(4) $$F\left(\mu \right)=\frac{d\,\mu }{d\,t}=\mu \left(U_{\mu }-{\bar{U}}_{\mu }\right)=\mu \left(1-\mu \right)\text{[}\rho v(R12+B1$$

-C10)+ρ(C10-R12-K-B1)+v(-R12-B1)

+W3+B1]

The expected benefits of an active participation strategy for the resident group are:


(5) $$U_{v}=\mu \,\rho \,\left(R\,11+R\,12+R\,13+R\,14-C\,11\right)+\mu \,\left(1-\rho \right)$$

(R11+R12+R13+R14-C11)+(1-μ)ρ(R11

+R13+R14-C11)+(1-μ)(1-ρ)(R11+R13

+R14-C11)=μR12+R11+R13+R14-C11

The expected benefits of a negative participation strategy for the resident group are:


(6) $$U_{1-v}=\mu \rho \left(R11+R14-D2\right)+\mu \left(1-\rho \right)(R11$$

+R14-D2)+(1-μ)ρ(R11+R14-D2)+(1-μ)

(1-ρ)⁢(R⁢11-D⁢2)=μ⁢ρ⁢(-R⁢14)+μ⁢R⁢14+ρ⁢R⁢14

+R⁢11-D⁢2

Therefore, the average expected benefits for residents choosing a mixed strategy of active or negative participation are:


(7) $${\bar{U}}_{v}=v\,U_{v}+\left(1-v\right)\,U_{1-v}$$

The replication dynamic equation for residents’ behavioral strategy is:


(8) $$F\left(v\right)=\frac{d\,v}{d\,t}=v\left(U_{v}-{\bar{U}}_{v}\right)=v\left(1-v\right)[\mu \rho R14+\mu (R12$$

-R14)-ρR14+R13+R14-C11+D2]

The expected benefits of community planners adopting an active promotion strategy are:


(9) $$U_{\rho }=\mu \,v\,\left(K+R\,16+R\,17+R\,18-C\,12\right)+\mu \,\left(1-v\right)$$

(K+R16+R17+R18-C12-D3)+(1-μ)v(R16

+R17+R18-C12)+(1-μ)(1-v)(R16+R17+

R18-C12-D3)=μK+vD3+R16+R17

+R⁢18-C⁢12-D⁢3

The expected benefits of a non-promotion strategy for community planners are:


(10) $$U_{1-\rho }=\mu \,v\,R\,15+\mu \,\left(1-v\right)\,R\,15+\left(1-\mu \right)\,v\,R\,15\,\left(1-\mu \right)$$

(1-v)⁢R⁢15=R⁢15

Therefore, the average expected benefits for community planners who choose a mixed strategy of active promotion or no promotion are:


(11) $${\bar{U}}_{\rho }=\rho \,U_{\rho }+\left(1-\rho \right)\,U_{1-\rho }$$

The replication dynamic equation for the behavioral strategies of community planners is:


(12) $$F\left(\rho \right)=\frac{d\,\rho }{d\,t}=\rho \left(U_{\rho }-{\bar{U}}_{\rho }\right)=\rho \left(1-\rho \right)[\mu K+vD3+R16$$

+R17+R18-C12-D3-R15]

To simplify subsequent calculations, let:


(13) $$\left\{ \begin{array}{c} R\,12+B\,1-C\,10=n\,1\\ C\,10-R\,12-K-B\,1=n\,2\\ -R\,12-B\,1=n\,3\\ W\,3+B\,1=n\,4\\ R\,12-R\,14=n\,5\\ R\,13+R\,14-C\,11+D\,2=n\,6\\ R\,16+R\,17+R\,18-C\,12-D\,3-R\,15=n\,7 \end{array}\right.$$

This results in a three-dimensional dynamical system N:


(14) $$\left\{ \begin{array}{c} F\,\left(\mu \right)=\frac{d\,\mu }{d\,t}=\mu \,\left(1-\mu \right)\,\left(\rho \,v\,n\,1+\rho \,n\,2+v\,n\,3+n\,4\right)\\ F\,\left(v\right)=\frac{d\,v}{d\,t}=v\,\left(1-v\right)\,\left(\mu \,\rho \,R\,14+\mu \,n\,5-\rho \,R\,14+n\,6\right)\\ F\,\left(\rho \right)=\frac{d\,\rho }{d\,t}=\rho \,\left(1-\rho \right)\,\left(\mu \,K+v\,D\,3+n\,7\right) \end{array}\right.$$

### Stabilization strategies of stakeholders


(15) $$F^{\prime }\left(\mu \right)=\left(1-2\mu \right)\text{[}\rho v\left(R12+B1-C10\right)+\rho (C10-R12$$

-K-B1)+v(-R12-B1)+W3+B1]

Let *F*(μ) = 0, then $\rho =\frac{\nu \,\left(R\,12+B\,1\right)-W\,3-B\,1}{\nu \,\left(R\,12+B\,1-C\,10\right)+C\,10-R\,12-K-B\,1}$, a dividing line in the evolution of stabilization strategies for grassroots government. When $\rho >\frac{\nu \,\left(R\,12+B\,1\right)-W\,3-B\,1}{\nu \,\left(R\,12+B\,1-C\,10\right)+C\,10-R\,12-K-B\,1}$, μ = 0, *F*′(μ) > 0; μ = 1, *F*′(μ) < 0. Therefore, when $\rho >\frac{\nu \,\left(R\,12+B\,1\right)-W\,3-B\,1}{\nu \,\left(R\,12+B\,1-C\,10\right)+C\,10-R\,12-K-B\,1}$, μ = 1 is an evolutionary stability point for grassroots government, the evolutionary stabilization strategy of grassroots government is to incentivize.

When $\rho <\frac{\nu \,\left(R\,12+B\,1\right)-W\,3-B\,1}{\nu \,\left(R\,12+B\,1-C\,10\right)+C\,10-R\,12-K-B\,1}$, μ = 0, _*F’(μ)<0*_; μ = 1, *F*′(μ) > 0.

When $\rho <\frac{\nu \,\left(R\,12+B\,1\right)-W\,3-B\,1}{\nu \,\left(R\,12+B\,1-C\,10\right)+C\,10-R\,12-K-B\,1}$, and μ = 0 is an evolutionary stabilization point for grassroots government, which has a strategy of no incentive.


(16) $$F^{\prime }\left(v\right)=\left(1-2v\right)[\mu \rho R14+\mu \left(R12-R14\right)-\rho R14$$

+R13+R14-C11+D2]

Let *F*(ν) = 0, then $\mu =\frac{\rho \,R\,14+C\,11-R\,13-R\,14-D\,2}{\rho \,R\,14+R\,12-R\,14}$, is the dividing line for the evolutionary stabilization strategy of the residents. When $\mu >\frac{\rho \,R\,14+C\,11-R\,13-R\,14-D\,2}{\rho \,R\,14+R\,12-R\,14}$, ν = 0, *F*′(ν) > 0; ν = 1, *F*′(ν) < 0, so $\mu >\frac{\rho \,R\,14+C\,11-R\,13-R\,14-D\,2}{\rho \,R\,14+R\,12-R\,14}$时, ν = 1 is the point of evolutionary stability of residents’ behavioral strategies, and the evolutionary stability strategy is active participation. When $\mu <\frac{\rho \,R\,14+C\,11-R\,13-R\,14-D\,2}{\rho \,R\,14+R\,12-R\,14}$, ν = 0, *F*′(μ) < 0; ν = 1, *F*′(ν) > 0. Therefore, when $\mu <\frac{\rho \,R\,14+C\,11-R\,13-R\,14-D\,2}{\rho \,R\,14+R\,12-R\,14}$, ν = 0 is the point of evolutionary stability for the residents and their evolutionary stability strategy is passive participation.


(17) F(ρ)=(1-2ρ)[μK+vD3+R16+R17

+R18-C12-D3-R15]

Let *F*(ρ) = 0, then $\mu =\frac{C\,12+D\,3+R\,15-R\,16-R\,17-R\,18-\nu \,D\,3}{K}$ is a dividing line in the evolution of stable strategies for the behaviors of community planners.

When $\mu >\frac{C\,12+D\,3+R\,15-R\,16-R\,17-R\,18-\nu \,D\,3}{K}$, ρ = 0, *F*′(ρ) > 0; ρ = 1, *F*′(ρ) < 0. Therefore, when $\mu >\frac{C\,12+D\,3+R\,15-R\,16-R\,17-R\,18-\nu \,D\,3}{K}$, ρ = 1 is an evolutionary stabilization point for community planners and evolutionary stabilization strategies for active promotion.

When $\mu <\frac{C\,12+D\,3+R\,15-R\,16-R\,17-R\,18-\nu \,D\,3}{K}$, ρ = 0, *F*′(ρ) < 0; ρ = 1, *F*′(ρ) > 0. Therefore, when $\mu <\frac{C\,12+D\,3+R\,15-R\,16-R\,17-R\,18-\nu \,D\,3}{K}$, ρ = 0 is the point of evolutionary stability for community planners, and the evolutionary stability strategy for community planners is not to promote.

### Analysis of system evolutionary stability points

Let *F*(μ) = *d*μ/*dt* = 0,*F*(*v*) = *dv*/*dt* = 0,*F*(ρ) = *d*ρ/*dt* = 0, then *E*1(0,0,0),*E*2(0,1,0),*E*3(0,1,1), *E*4(1,0,1),*E*5(1,0,0),*E*6(1,1,0),*E*7(0,0,1), and *E*8(1,1,1) are pure strategic partial equilibrium points for system N, and the mixed strategy partial equilibrium point is *E*9(μ*,v*,ρ*), 0 < μ* < 1, 0 < v* < 1, 0 < ρ* < 1


(18) $$\left\{ \begin{array}{c} \rho \,v\,n\,1+\rho \,n\,2+v\,n\,3+n\,4=0\\ \mu \,\rho \,R\,14+\mu \,n\,5-\rho \,R\,14+n\,6=0\\ \mu \,K+v\,D\,3+n\,7=0 \end{array}\right.$$

According to Friedman’s theory, the Jacobian matrix J3 of the three-dimensional dynamical system N is obtained:


(19) $$J\,3=\left[\begin{array}{ccc} \frac{\partial F\,\left(\mu \right)}{\mu } & \frac{\partial F\,\left(\mu \right)}{\nu } & \frac{\partial F\,\left(\mu \right)}{\partial \rho }\\ \frac{\partial F\,\left(\nu \right)}{\mu } & \frac{\partial F\,\left(\nu \right)}{\nu } & \frac{\partial F\,\left(\nu \right)}{\partial \rho }\\ \frac{\partial F\,\left(\rho \right)}{\mu } & \frac{\partial F\,\left(\rho \right)}{\nu } & \frac{\partial F\,\left(\rho \right)}{\partial \rho } \end{array}\right]=$$

$$[\begin{array}{ccc} \left(1-2\,\mu \right)\,\left(\rho \,\nu \,n\,1+\rho \,n\,2+\nu \,n\,3+n\,4\right) & & \\ \nu \,\left(1-\nu \right)\,\left(\rho \,R\,14+n\,5\right) & & \\ \rho \,\left(1-\rho \right)\,K & & \end{array}$$

$$\begin{array}{cc} \mu \,\left(1-\mu \right)\,\left(\rho \,n\,1+n\,3\right)\; & \\ \left(1-2\,\nu \right)\,\left(\mu \,\rho \,R\,14+\mu \,n\,5-\rho \,R\,14+n\,6\right)\; & \\ \rho \,\left(1-\rho \right)\,D\,3\; & \end{array}$$

$$\begin{array}{cc} \mu \,\left(1-\mu \right)\,\left(\nu \,n\,1+n\,2\right)\; & \\ \nu \,\left(1-\nu \right)\,\left(\mu \,R\,14-R\,14\right)\; & \\ \left(1-2\,\rho \right)\,\left(\mu \,K+\nu \,D\,3+n\,7\right)\; & \end{array}]$$

Based on the Lyapunov stability condition, the eight pure strategies Nash equilibria of system N are brought into the Jacobi matrix to calculate the Eigenvalues of each equilibrium point and its evolutionary stability conditions, as shown in Tables 5, 6.

**TABLE 5:** Eigenvalues of the equilibrium points of the system N.

Equilibrium point	λ1	λ2	λ3	Progressive stability conditions
*E*1(0,0,0)	*n4*	*n6*	*n7*	*n*4 < 0,*n*6 < 0,*n*7 < 0
*E*2(0,1,0)	*n*3+*n*4	−*n*6	*D*3+*n*7	*n*3+*n*4 < 0,−*n*6 < 0,*D*3+*n*7 < 0
*E*3(0,1,1)	*n*1+*n*2+*n*3+*n*4	*R14-n6*	−*D*3−*n*7	*n*1+*n*2+*n*3+*n*4 < 0,*R*14−*n*6 < 0, −*D*3−*n*7 < 0
*E*4(1,0,1)	−*n*2-*n*4	*n*5+*n*6	−*K*-*n*7	−*n*2-*n*4 < 0,*n*5+*n*6 < 0,−*K*-*n*7 < 0
*E*5(1,0,0)	−*n*4	*n*5+*n*6	*K* + *n*7	−*n*4 < 0,*n*5+*n*6 < 0,*K* + *n*7 < 0
*E*6(1,1,0)	−*n*3−*n*4	−*n*5-*n*6	*K* + *D*3 + *n*7	−*n*3−*n*4 < 0,−*n*5-*n*6 < 0,*K* + *D*3 + *n*7 < 0
*E*7(0,0,1)	*n*2+*n*4	*n*6-*R*14	−*n*7	*n*2+*n*4 < 0,*n*6-*R*14 < 0,−*n*7 < 0
*E*8(1,1,1)	−*n*1+*n*2-*n*3-*n*4	−*n*5-*n*6	−*K*−*D*3−*n*7	−*n*1+*n*2-*n*3-*n*4 < 0,−*n*5-*n*6 < 0, −*K*−*D*3−*n*7 < 0

**TABLE 6:** Evolutionary stability conditions for the equilibrium point of the system N.

Equilibrium point	Evolutionary stability conditions
*E*1(0,0,0)	λ1=*W*3+*B*1 > 0, unstable point
*E*2(0,1,0)	*W*3−*R*12 < 0,−*R*13−*R*14+*C*11−*D*2 < 0,*R*16+*R*17+*R*18−*C*12−*R*15 < 0
*E*3(0,1,1)	−*R*12−*K* + *W*3 < 0,−*R*13+*C*11−*D*2 < 0,−*R*16−*R*17−*R*18+*C*12+*R*15 < 0
*E*4(1,0,1)	*R*12+*K*-*W*3-*C*10 < 0,*R*12+*R*13-*C*11+*D*2 < 0,−*K*-*R*16-*R*17-*R*18+*C*12+*D*3+*R*15 < 0
*E*5(1,0,0)	−*W*3-*B*1 < 0,*R*12+*R*13-*C*11+*D*2 < 0*K* + *R*16+*R*17+*R*18-*C*12-*D*3-*R*15 < 0
*E*6(1,1,0)	*R*12−*W*3 < 0,*C*11−*R*12−*R*13−*D*2 < 0,*K* + *R*16+*R*17+*R*18−*C*12−*R*15 < 0
*E*7(0,0,1)	*C*10−*R*12−*K* + *W*3 < 0,*R*13−*C*11+*D*2 < 0,*C*12+*D*3+*R*15−*R*16−*R*17−*R*18 < 0
*E*8(1,1,1)	*R*12+*K*−*W*3 < 0,*C*11−*R*12−*R*13−*D*2 < 0,−*K*−*R*16−*R*17−*R*18+*C*12+*R*15 < 0

As can be seen in Table 6, there are seven possible evolutionary stabilization strategies for the three-dimensional dynamical system N when the corresponding evolutionary stabilization conditions are met, namely *E2(0,1,0), E3(0,1,1), E4(1,0,1), E5(1,0,0), E6(1,1,0), E7(0,0,1)*, and *E8(1,1,1). E3(0,1,1)* is the ideal state of community micro-renewal in which residents participate in collaborative governance. However, in China, *E4(1,0,1)* and *E7(0,0,1)* are the general states of community micro-renewal projects at this stage, with a lack of resident participation. *E8(1,1,1)* is the ideal strategy for community micro-renewal (i.e., government incentives, active resident participation, active promotion by community planners). Therefore, this article next addresses the measures the government, residents, and community planners should take to make the game system N evolve and stabilize at E8(1,1,1) in the community micro-renewal process. Table 6 shows that the conditions for system N to reach evolutionary stability at *E8(1,1,1)* are: *R12+K−W3 < 0, C11−R12−R13−D2 < 0, C12+R15−K−R16−R17−R18 < 0*.

## Simulation analysis

### Evolutionary path analysis

**FIGURE 2:**
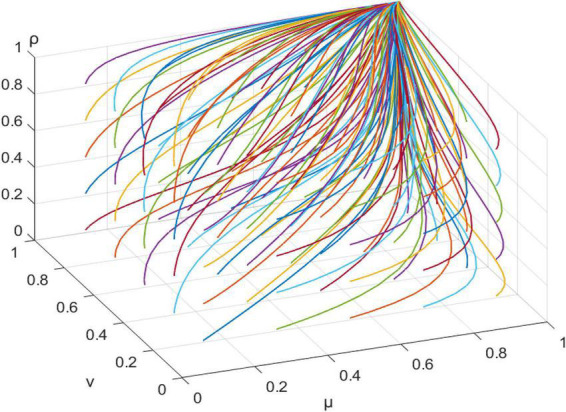
Evolutionary paths of the behavior of a three-party game with different initial values.

This article assigns values to each parameter, as shown in Table 7. MATLAB was used for simulation analyses, to obtain the evolutionary path of the three-party game behavior with different initial values, as shown in Figure 2.

**TABLE 7:** Simulation parameter settings.

Parameters	Value	Parameters	Value
*B1*	20	*R14*	20
*C10*	10	*R15*	15
*C11*	20	*R16*	20
*C12*	25	*R17*	20
*K*	10	*R18*	15
*W3*	30	*D2*	30
*R12*	10	*D3*	25
*R13*	15		

From Figure 2, when *R12+K−W3 < 0, C11−R12−R13−D2 < 0*, and *C12+R15−K−R16−R17 −R18 < 0*, μ, v, and ρ take different initial values, the system will reach the ideal evolutionary stable equilibrium *E(1,1,1)*. At this point, the government takes incentives, residents actively participate, and community planners actively contribute. In the context of China, during the community micro-renewal planning stage, the government needs to guide top-down community planning and construction through a high-level design. When community micro-renewal enters the implementation stage, the local government should be less involved and rely mainly on community planners and residents to achieve community micro-renewal goals.

### Parametric analysis

Herein, MATLAB was used to simulate the relevant model parameters and analyze the influence of each parameter on the evolutionary game behavior of three subjects in community micro-renewal. This article selected four primary parameters, *K, W3, R13*, and R16, for analysis. Initially, the probability of grassroots government, resident groups, and community planners choosing different strategies is set to 20%. Take the parameters in Table 5 as the reference group. When a parameter changes, the other values remain unchanged. This analysis follows.

#### Impact of economic subsidies provided by grassroots government (K) on evolutionary trajectories

**FIGURE 3:**
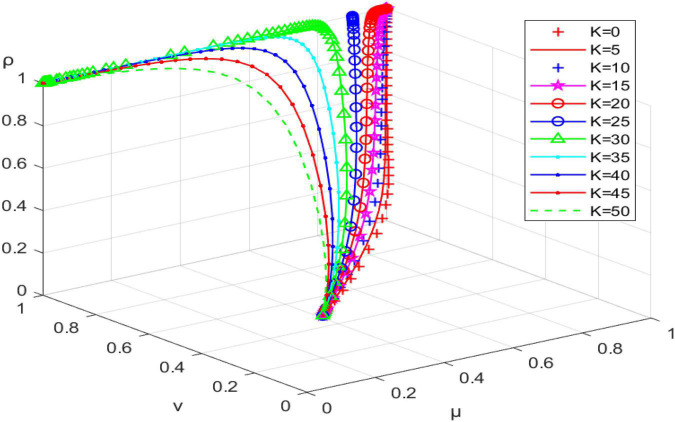
System evolution path when the government provides financial subsidies (K) to community planners.

Let K take 0, 5, 10, 15,…, 50, and we obtain the simulation results shown in Figure 3. When K is 0–20, the system reaches the ideal evolutionary stability (incentive, active participation, active promotion). The probability of community planners choosing the “active promotion” strategy increases as the value of K increases. At a *K* value of 25, the system is not evolutionarily stable. As K continues to increase, the system will reach evolutionary stability at (no incentive, active participation, active promotion), where excessive financial subsidies make the burden on the government too high, and the government chooses the no incentive strategy. Therefore, the government should provide appropriate financial subsidies to community planners to effectively promote implementation of community micro-renewal projects.

#### Impact of resident management achievement (R13) on system evolution

**FIGURE 4:**
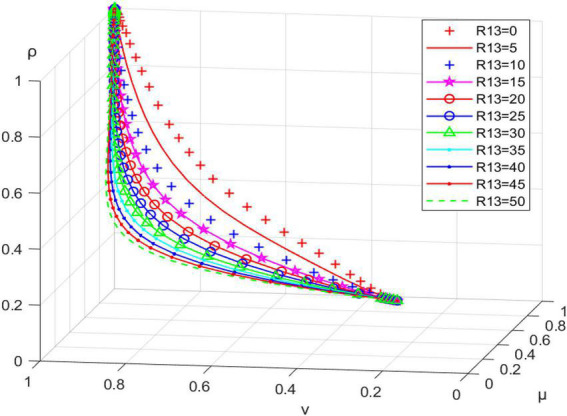
Impact of residents’ sense of managerial achievement (R13) on evolutionary path.

Letting R13 be 0, 5, 10, 15,…, 50, we can get the simulation results in Figure 4. As the residents’ sense of achievement increases, the system reaches the ideal stable state more quickly. Therefore, the grassroots government can gradually delegate community management governance to residents to enhance their sense of ownership and responsibility, conducive to promoting positive interactions among stakeholder groups and then sustainably promoting the construction and implementation of community micro-renewal projects.

#### Impact of community planners’ project income (R16) on system evolution path

**FIGURE 5:**
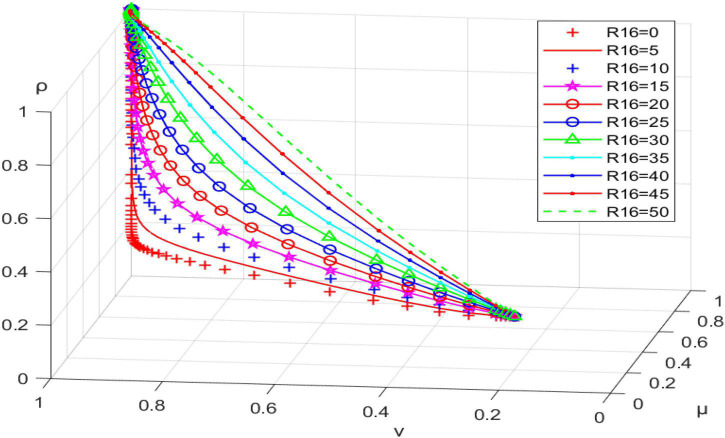
Impact of community planners’ project income (R16) on system evolution path.

Letting R16 be 0, 5, 10, 15….50, we obtain the results shown in Figure 5. As the income of community planners’ projects increases, the system reaches the ideal evolutionary steady state at a faster rate. Therefore, government departments should develop an effective system for distributing community planners’ earnings to guarantee a reasonable financial income to improve the current situation. Community planners are mainly driven by personal sentiments, increasing their enthusiasm for participating in community governance.

When the conditions R12+K−W3 < 0, C11−R12 −R13−D2 < 0, and C12+R15−K−R16−R17−R18 < 0 are satisfied, the tripartite evolutionary game system will reach the desired evolutionary stability (incentive, active participation, active promotion). Financial subsidy (K) of the grassroots government to the community planner has a positive effect on the behavioral choice of the latter and, to some extent, a negative impact on the behavioral intention of the grassroots government. It facilitates the tripartite game system reaching ideal evolutionary equilibrium within reasonable limits. Higher levels of incentives (W3) have a direct positive effect on the behavioral choices of the grassroots government and an indirect positive impact on the behavioral intentions of community planners and residents; the greater the W3, the faster the tripartite evolutionary game system reaches ideal evolutionary stability. The sense of achievement (R13) gained by residents’ participation in the follow-up management of the micro-renewal project positively affects their strategy choice. It can effectively promote the tripartite game system reaching ideal evolutionary equilibrium. The benefits gained by community planners by promoting community micro-renewal (R16) can effectively promote their positive behavioral choices. They can also contribute to the desired evolutionary equilibrium of the tripartite game system.

## Discussion

The analyses herein show that grassroots governments must improve the community environment to meet residents’ needs for a better life and to enhance political performance and social prestige. Resident groups are concerned with improving their living environment and quality of life, and hope that their diverse needs can be met. Personal sentiment is the primary motivation for community planners to participate in micro-renewal. Their interests are not only financial gain from completing the project but the need to enhance their professional competence, visibility, and industry reputation and meet academic research needs. Community planners can provide professional planning and design and regeneration solutions, act as a “bridge” between the government and residents, and identify the intrinsic needs of residents so that community micro-renewal can genuinely solve practical problems.

According to the evolutionary game and simulation analysis, the theoretical conditions for the stakeholders of community micro-renewal to reach the ideal evolutionary equilibrium are *C10+R12+K−W4−W5 < 0, C12−R12−R13−D2 < 0, C13+R15−K−R16−R17−R18 < 0*. The parameters *C10, K, W3, R13*, and *R16*, will impact the behavioral strategies of the grassroots government, community residents, and community planners. These findings support the following recommendations.

### Grassroots government

The grassroots government should increase its efforts to publicize community micro-renewal. Apart from regular project promotion meetings and seminars, it can conduct on-site visits to successful projects to tell residents more about the benefits of community micro-renewal. It is also essential to conduct thorough research on community issues to accurately conceptualize residents’ needs. Simultaneously, higher authorities can mobilize grassroots governments to promote community micro-renewal, such as by including the effectiveness of community micro-renewal in assessments of grassroots governments and developing an incentive system.

The role of the grassroots government in community micro-renewal should change from “leading” to “guiding,” with the top-level design and overall system planning accomplished in the early project stage. The task’s structure, construction, and management are then delegated to community planners and residents during the implementation stage. The government should provide essential policy support and resources. The grassroots government should provide incentives for community planners during the early community renewal stages. By designing relevant systems, community planners’ rights, status, and positive benefits should be safeguarded to promote sustainable community micro-renewal development.

Although the urban and rural planning law includes community planning into the legal scope of urban and rural planning, community planning has not been included in the working mechanism of the legal urban planning system. Therefore, the government should improve the relevant system.

### Community planner

Though the composition of community planners and the focus of their work vary regionally, their core objectives are to coordinate the demands of community stakeholders, from a community-based perspective, and to alleviate conflicts of interest by effectively enriching public participation channels.

As the core for community regeneration planning and design, community planners must respond to the government’s call to apply their professional skills to solve social problems and actively assume social responsibility. Community planners should mobilize and organize the public through various activities to attract residents’ participation and muster their enthusiasm. They should take a humanistic approach, improving the community’s physical space, gaining a deeper understanding of residents’ needs, and making professional amendments based on their renewal ideas to realize their aspirations to the extent possible. In addition, community planners should provide residents with specific professional knowledge training to lower the barriers to their participation and foster involvement in follow-up management of community micro-renewal.

### Community residents

Residents should change their past attitude of indifference to community affairs and instead respond actively to calls by government departments. They should also enhance their sense of ownership, take the initiative to identify community problems, and offer advice and suggestions for community micro-renewal. After the renewal is complete, they should take responsibility for community management and maintain the micro-renewal results. They should also continue to improve their cultural literacy and actively participate in training activities organized by community organizations and planners. Community micro-renewal success cannot be achieved without all parties’ collaborative contributions and governance.

## Data availability statement

The original contributions presented in this study are included in the article/supplementary material, further inquiries can be directed to the corresponding author.

## Author contributions

DW designed the research framework and methodology. MW constructed the game model and simulation analyses. JQ was responsible for the literature analysis and strategy research. YF edited the manuscript. All authors confirmed and approved all sections of the final manuscript.
